# Visitor’s Experiences of an Evidence-Based Designed Healthcare Environment in an Intensive Care Unit

**DOI:** 10.1177/1937586720943471

**Published:** 2020-07-31

**Authors:** Fredrika Sundberg, Isabell Fridh, Berit Lindahl, Ingemar Kåreholt

**Affiliations:** 1 Faculty of Caring Science, Work Life and Social Welfare, 1802 University of Borås, Sweden; 2 Institute of Gerontology, School of Health and Welfare, 4161 Jönköping University, Sweden

**Keywords:** academic research, family-centered care, intensive care unit (ICU), interior design, access to nature, design research, evidence-based design (EBD), nursing research, patient-/person-centered care, patient room design

## Abstract

**Objectives:**

The objective of the research was to study the visitors’ experiences of different healthcare environment designs of intensive care unit (ICU) patient rooms.

**Background:**

The healthcare environment may seem frightening and overwhelming in times when life-threatening conditions affect a family member or close friend and individuals visit the patient in an ICU. A two-bed patient room was refurbished to enhance the well-being of patients and their families according to the principles of evidence-based design (EBD). No prior research has used the Person-centred Climate Questionnaire—Family version (PCQ-F) or the semantic environment description (SMB) in the ICU setting.

**Methods:**

A sample of 99 visitors to critically ill patients admitted to a multidisciplinary ICU completed a questionnaire; 69 visited one of the two control rooms, while 30 visited the intervention room.

**Results:**

For the dimension of everydayness in the PCQ-F, a significantly better experience was expressed for the intervention room (*p* < .030); the dimension regarding the ward climate general was also perceived as higher in the intervention room (*p* < .004). The factors of pleasantness (*p* < .019), and complexity (*p* < 0.049), showed significant differences favoring the intervention room in the SMB, with borderline significance on the modern factor (*p* < .061).

**Conclusion:**

Designing and implementing an enriched healthcare environment in the ICU setting increases person-centered care in relation to the patients’ visitors. This could lead to better outcomes for the visitors, for example, decreasing post-traumatic stress disorder symptoms, but this needs further investigations.

## Background

### Visiting the Intensive Care Unit (ICU)

The environment in ICUs is dominated by sophisticated technology due to the seriousness of admitted patients’ conditions, which are often life-threatening. In times of stress and crisis, visiting relatives are exposed to an environment that may seem frightening and overwhelming to them. The alien environment, with its advanced monitoring and aggressive treatments of critically ill patients, is harsh for the family members (Imanipour et al., 2019; Ruckholdt et al., 2019; Turner-Cobb et al., 2016). Experiences like anxiety, sadness, depression, and fatigue in family members of ICU patients have been reported repeatedly in prior studies (Apple, 2014; Celik et al., 2016; Day et al., 2013). These stressful experiences can sometimes develop into more persistent conditions such as post-traumatic stress disorder (Petrinec & Daly, 2016). Despite the unfriendly environment, the need and desire to visit and be close to the critically ill patient has had the same high priority among family members for the last 40 years (Jacob et al., 2016; Plakas et al., 2014). Despite this, many ICUs have restricted visiting hours. Nevertheless, the ongoing trend is to shift toward open visiting hours, with more satisfied family members as a result (Chapman et al., 2016). Open visiting hours represent one way of implementing person-/family-centered care in the ICU (Coombs et al., 2017; Davidson et al., 2017).

### Person-Centered Care (PCC)

In healthcare, from a medical perspective, patients can be seen by their diagnoses, illnesses, or body parts to treat them rather than see them holistically as people. In contrast, PCC emphasizes the significance of recognizing the person behind the patient, as a human being with meaning, will, emotions, and needs (Ekman et al., 2011; Mounier, 1952; World Health Organization, Regional Office for the Western Pacific, 2007). By promoting humane and holistic ways, the goal for PCC is improving outcomes for persons, families, health workers, organizations, and health systems. The values and preferences expressed by individuals guide all aspects of healthcare in PCC. This is accomplished via a relationship among individuals, their close ones, and all relevant contributors (“PCC: A Definition and Essential Elements,” 2016). This paradigm shift from the medical point of view to the holistic view of PCC may reestablish harmony and balance for individuals as well as the harmony and affinity between people and their environment (World Health Organization, Regional Office for the Western Pacific, 2007).

PCC has developed into the wider concept of family-centered care (FCC). In intensive care settings, FCC has been defined as a respectful and responsive approach to individual families’ needs and values (Davidson et al., 2017). The recognition of FCC is considered a crucial part of high-quality care in ICUs, and implementation does not require special equipment or significant financial investments (Gerritsen et al., 2017). Although an improved design and construction of the ICUs may facilitate FCC, it may also cause disturbance for the staff (Rippin et al., 2015). The difficulty in implementing PCC in healthcare is not that the staff are skeptical of the concept but rather that they are already under the impression they are working with a PCC approach even though they are not (Ekman et al., 2011; Santana et al., 2018).

### The Design of ICUs

The environment in ICUs can affect patients, their visiting family members, and staff by either increasing or decreasing their levels of distress. Evidence-based design (EBD) has evolved as a research field where the effects of architecture on health environments are in focus (Ulrich et al., 2010). The design of ICUs has not had the same progress as the medical technology has, and therefore, new equipment is placed where there is a free space rather than being integrated into the design. However, there have been attempts to implement an enriched environment in intensive care. It has been found that family members visiting hospital gardens show decreased distress (Ulrich et al., 2019). Implementing access to nature during the ICU stay has positive effects for patients, families, and staff (Minton & Batten, 2016; Sundberg et al., 2017).

Family members of critically ill patients play a crucial part in the team around the patient and are pivotal in the recovery and terminal phases. However, there is a risk that they may feel out of place due to the high-technological and foreign surroundings of the ICU or neglected by the staff due to their workload around the patient care. If the visitors have a good experience of the environment, they will feel more part of the healthcare team (e.g., if the environment feels very medical and intense for the visitors, they may feel uncomfortable and not offer their insight in the care of their loved ones, yet previous research has shown that support of loved ones leads to better outcomes (Gooding et al., 2012). Thus, if they feel they are in a welcoming and comfortable environment, they could feel like they are part of the healthcare team, and it could also lead to decreased stress on themselves as well.

Therefore, it is important to gain knowledge about how visitors at ICUs experience the overall ward climate. This study attempts to give family members a voice to describe the perceived healthcare environment that surrounds the critically ill patient.

## Aim

The aim of the research was to study visitors’ experiences of different healthcare environment designs of ICU patient rooms.

## Method

### Setting

**Figure 1.**
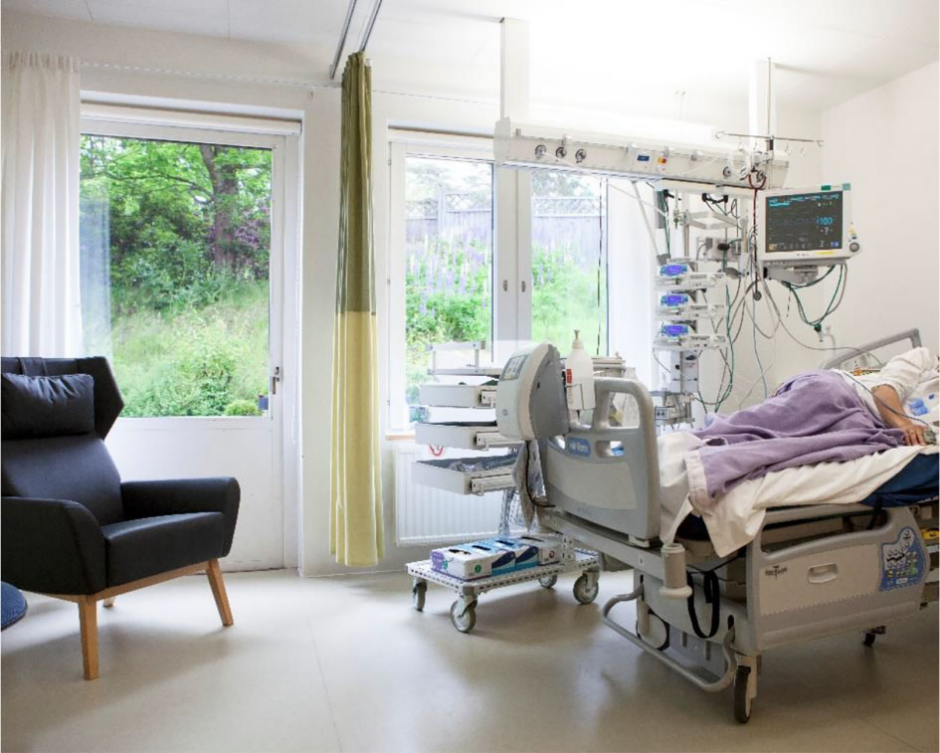
Intervention room. ©Lindahl

**Figure 2.**
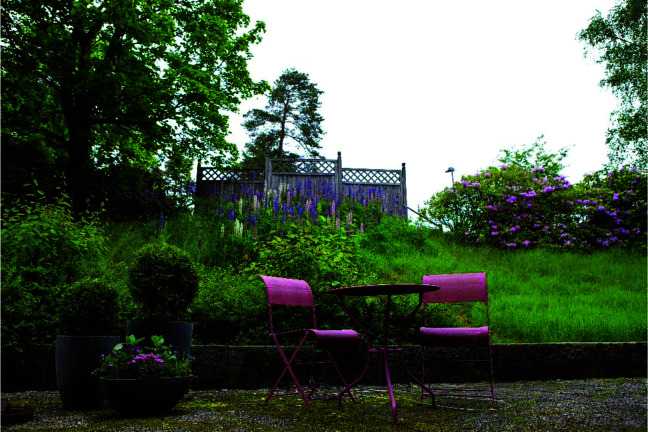
Patient’s and visitor’s view and access to nature in the intervention room. ©Lindahl

**Figure 3.**
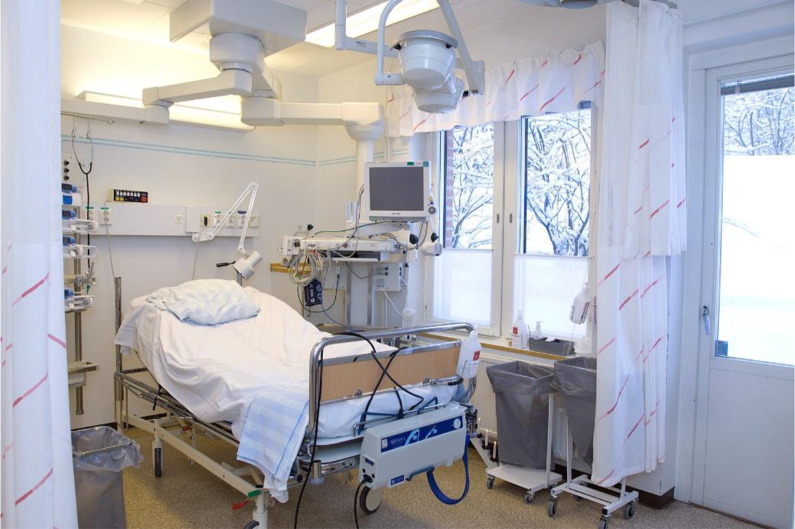
Control room. ©Lindahl

The study was executed at a 395-bed hospital in Sweden, which comprises a multidisciplinary 10-bed ICU with 650 enrolments yearly. In 2010, a two-bed intensive care patient room was refurbished through multidisciplinary teamwork (B. Lindahl & Bergbom, 2015), according to the principles of EBD and considering the guidance for complex intervention research (Craig et al., 2008). The patient room was completely refurbished, although this was done within the existing area. Acoustic panels were built into the walls and ceiling, and new flooring was installed. In addition, pendulums with electrical sockets and medical gas supplies and cyclic lights—to preserve the patient’s circadian rhythm—were installed. Calming colors were brought to the room by ecological textiles in curtains, bedsheets, and blankets for the patients (see Figure 1). The linens for the intervention room were chip-marked, and they had their own laundry bags to distinguish them from the bed linen that was used in the other ordinary patient (control) rooms. During the research period, the research team supplied additional bed linen as needed to maintain the intervention manipulation. All furnishings were constructed of ecological materials, while it was ensured that the furniture for the visitors was comfortable. Patients and their visitors had access to nature via a window and door leading onto a patio in the greenery (see Figure 2), with furniture and seasonal plants (B. Lindahl & Bergbom, 2015). Two rooms, which were identical to how the intervention room was previously designed, were used as control rooms. The control rooms were situated next to the intervention room. The control rooms had frosted glass to prevent outsiders from seeing the patients, but this also limited patients and their family members being able to see the outside from the rooms (see Figure 3). Patients and their visiting family members in the control rooms also had access to a patio but with no furniture or planted flowers. There were no refurbishments in the ICU during the data collection period.

### Questionnaires

#### The Person-centred Climate Questionnaire—Family version (PCQ-F)

The PCQ-F (J. Lindahl et al., 2015), which evaluates the dimensions of *safety*, *everydayness*, and *hospitality* of the psychosocial care climate, was used in this study. According to the researchers who developed the questionnaire, different requirements need to be met for sensing the three dimensions. A climate of safety can be perceived when family members find staff available and approachable, viewing their actions as competent and comprehensible. It is crucial for safety that, in addition to being clean, the environment sanctions space for privacy and interaction with others. Because many of the questionnaire items focus on the staff and not the built environment, we split the dimension into safety, *staff*, and *ward climate safety* (see Table 1). A climate of everydayness appears when patients and families feel acquainted to the surrounding environment and sense tranquility and when the place offers positive distractions for patients and family members to divert their thoughts from illness and treatment. Finally, a climate of hospitality is perceived when the environment communicates a sense of welcoming and feeling that the care and treatment appear to exceed expectations. It is essential for patients and family members to be seen, met, and welcomed, and furthermore, to sense generosity from the staff (J. Lindahl et al., 2015). The questionnaire contained questions on the dimensions with 6-point Likert-type scales (1 = *no, I disagree completely*, 6 = *yes, I agree completely*). An example of an item: a place that has something nice to look at (e.g., views, artwork).

**Table 1.** Ward Climate Questionnaire (PCQ-F).

	Ward Climate								
	Model 1			Model 2			Model 3		
	β Coefficient	*p* Value	CI	β Coefficient	*p* Value	CI	β Coefficient	*p* Value	CI
Ward climate, general (*x* ^a^)	.547	.017	[0.099, 0.994]	.651	.007	[0.181, 1.120]	.730	.004	[0.235, 1.225]
Ward climate, safety (*y*)	.558	.059	[−0.022, 1.138]	.583	.070	[−0.047, 1.213]	.563	.090	[−0.087, 1.214]
Everydayness (*z*)	.367	.100	[−0.070, 0.804]	.471	.044	[0.014, 0.929]	.533	.030	[0.050, 1.015]
	Ward Climate Staff								
Ward climate, staff (*w*)	.152	.511	[−0.302, 0.606]	.189	.436	[−0.286, 0.664]	.093	.714	[−0.402, 0.587]
Safety, staff (*v*)	.110	.672	[−0.397, 0.616]	.116	.679	[−0.433, 0.665]	.074	.799	[−0.497, 0.645]
Hospitality (*u*)	.284	.211	[−0.161, 0.730]	.335	.159	[−0.131, 0.801]	.294	.238	[−0.194, 0.783]

*Note*. Model 1: Crude differences between intervention room and control rooms. Model 2: Age, sex, and relationship to the patient were added. Model 3: Number of visits and whether the patient changed rooms during the intensive care unit stay were added. PCQ-F = Person-centred Climate Questionnaire—Family version; CI = confidence interval.

^a^ Indicates the number items included in the index.

#### The semantic environment description (SMB)

The SMB (*Swedish—Semantisk miljöbeskrivning*) is a structured method used for describing the impression of an architectural environment, where the environment can be interior, exterior, or simulated (Kuller et al., 1991). The SMB method is a questionnaire containing 36 adjectives measuring the overall impression of an environment. To identify how well each adjective agrees with the respondents’ perception of the environment, the questionnaire contains scales in the range of 1–7 (1 = *slightly*, 7 = *very*). The adjectives are clustered into the eight following factors: *pleasantness*, *complexity*, *unity*, *potency*, *social status*, *enclosedness*, *affection*, and *originality*. Due to the development of language and society, we have chosen to rename the factor *affection* as *modern* (Figure 1). No other changes were made.

### Data Collection

The questionnaires were distributed to visitors over the age of 18, such as family members and close friends, when they were visiting the critically ill patients cared for in the ICU. The staff and one of the researchers (F. S.) managed the distribution. The questionnaires were stored in the patient rooms, and one of the researchers (F. S.) regularly checked that there were always questionnaires to be handed out. F. S. also ensured to collect the ones that were answered and store them in a sealed envelope. The staff were instructed to invite every visitor over the age of 18 to participate when they estimated the situation was suitable (respecting the life-threatening condition of the patient). Visitors participating in this study were asked to complete the questionnaires while in the patient room (either in the intervention room or one of the two control rooms). This was done to ensure the participants were present in the real environment being evaluated. The number of previous visits to the ICU varied from 0 to 22 as described in Table 2. All the data come from participant responses to the questionnaire. The researchers had no direct access to any medical records. The data collection took place during November 2015–April 2019, and a total of 104 questionnaires were collected. Five questionnaires were excluded due to missing information about which room the visitor had visited.

**Table 2.** Descriptions of SMB Factors and Adjectives Included in Each Factor (Küller, 1991).

Factors	Descriptions	Items
Pleasantness	The degree of pleasantness, beauty, and security in the environment	Stimulating, secure, idyllic, good, pleasant, ugly (−), boring (−), brutal (−)
Complexity	The degree of variation, intensity, contrast, and abundance in the environment	Varied, lively, composite, subdued (−)
Unity	The fit of the different parts of the environment into a coherent whole	Functional, of pure style, consistent, whole
Enclosedness	A sense of spatial enclosure	Closed, demarcated, open (−), airy (−)
Potency	An expression of power latent in the environment	Masculine, potent, feminine (−), fragile (−)
Social Status	Evaluation in socioeconomic terms and in terms of maintenance	Expensive, well-kept, lavish, simple (−)
Modern ^a^	An age aspect as well as a quality of recognition	Modern, new, timeless (−), aged (−)
Originality	The unusual and surprising in the environment	Curious, surprising, special, ordinary (−)

*Note*. (–) indicates reverse coded.

^a^ Originally called affection.

## Ethical Considerations

The data collection was authorized by the Regional Ethical Review Board in Gothenburg, Sweden (No. 695-10), and institutional permission was obtained from the ward manager. The study followed the principles of ethical research as stated in the Declaration of Helsinki (World Medical Association, 2013) by assessing the risk, burdens, and benefits for the study participants. The front page of the questionnaires, which was removable for the participants, contained information about the study and had the researchers’ contact information if any questions or concerns arose. The information leaflet served as informed consent, as participation was voluntary. The questionnaires were answered anonymously, and there was no information from the participants that could link the answers to them or to any patient. The characteristics of the participants consisted of age, sex, relationship to the critically ill patient, and information about how many visits the participants had made and how long they had been in the room before conducting the questionnaire.

### Dependent Variables

A number of regression analyses were done on the dependent variables. The dependent variables were items from the different dimensions on the questionnaires: Ward climate—general, ward climate—safety, everydayness, ward climate—staff, safety—staff, and hospitality were from the PCQ-F, and the factors from the SMB included the following: pleasantness, complexity, unity, enclosedness, potency, social status, modernity, and originality. The dimensions on the PCQ-F were based on 3–10 items (see Table 3) answered on 6-point Likert-type scales, while the factors on the SMB were on four or eight adjectives answered on 7-point Likert-type scales (see Table 2).

**Table 3.** Results From the PCQ-F and SMB.

	Control Rooms *n* = 69	Intervention Room *n* = 30	*p* for Difference
PCQ-F	^a^	^a^	^b^
Ward climate, general	5.00	5.38	.006
Ward climate staff	5.42	5.49	.474
Factors in SMB	^c^	^c^	^d^
Pleasantness	4.91	5.25	.019
Complexity	3.52	3.31	.049
Unity	5.10	5.21	.359
Enclosedness	3.97	4.00	.862
Potency	3.87	4.20	.124
Social status	5.20	5.30	.791
Modernity	4.50	4.84	.061
Originality	3.65	3.56	.461

*Note*. PCQ-F = Person-centred Climate Questionnaire—Family version; SMB = semantic environment description.

^a^ Mean values. ^b^ *p* Values based on *t* test. ^c^ Adjusted mean values based on linear regressions controlled for age, sex, relation to the patient, number of visits, and whether patient had changed rooms during intensive care unit stay. ^d^ *p* Values based on ordinal probit regressions.

### Analyses

The dimensions in PCQ-F were analyzed with ordinal probit models. The results are presented as β coefficients and *p* values from three models: Model 1, crude differences between intervention room and control rooms; Model 2, additionally controlled for age, sex, and relationship to the patient; and Model 3, additionally controlled for the number of visits and whether the patient changed rooms during the ICU stay. The PCQ-F had item nonresponse (*n* = 0–8). Multiple imputations with fully conditional specification, including all PCQ-F items, were used to impute missing values. The data were analyzed twice, both with and without imputed missing data, to control for potential bias from partial nonresponse, which may have limited the results. However, there were no differences in the results where the presented findings in this study were calculated on nonimputed data. The items that concerned the staff and ward climate in general were analyzed separately because this study’s main focus was on ward climate (see Tables 1 and 4).

**Table 4.** Characteristics of the Visitors of the Control and Intervention Rooms.

	Control Rooms *n* = 69	Intervention Room *n* = 30	*p* for Difference ^a^
	% (*n*) ^b^	% (*n*) ^b^	
Female	59 (41)	70 (21)	.317
Visitors of patients who changed room during intensive care unit stay	39 (27)	63 (19)	.062
Age (years), mean (min–max)	49 (18–84)	49 (20–77)	.972 ^c^
Relationship			
Spouse/cohabitant	28 (19)	27 (8)	.929
Parent	14 (10)	20 (6)	.556 ^d^
Child	23 (16)	33(10)	.292
Other	35 (24)	20 (6)	.141
Number of previous visits, mean, median (min–max)	4.1, 2.0 (0–22)	3.3, 1.0 (0–11)	.400 ^c^
Length of visit (hours), mean, median (min–max)	1.4, 1.0 (0.05–9.0)	2.5, 1.0 (0.15–21.0)	.125 ^e^

^a^ Based on χ^2^ tests unless otherwise stated. ^b^ % (*n*) unless otherwise stated. ^c^ *t* Test. ^d^ Fisher’s exact test. ^e^ *p* Values based on binary logistic regressions with control versus intervention rooms as dependent variables.

**Figure 4.**
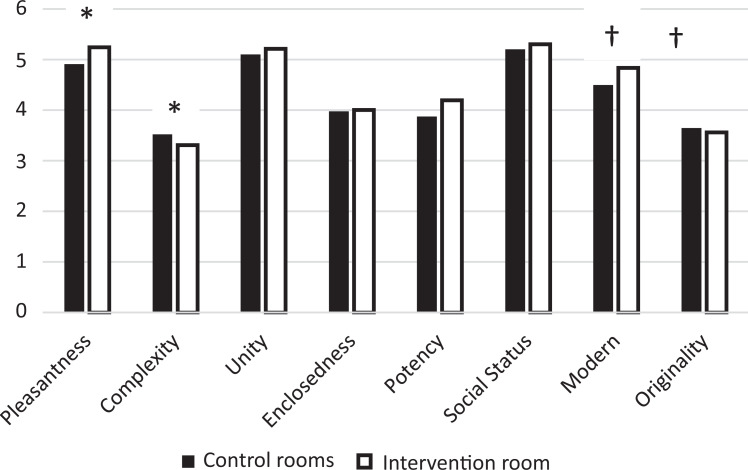
Semantic environment description in control rooms and intervention room. *Note*. Adjusted mean values based on linear regressions, *p* values based on ordinal probit analyses. The results are also presented in Table 4. *p < .05. †*p* < .10.

The factors in SMB are presented in Figure 4 as weighted mean values controlled for age, sex, relation to the patient, number of visits, and whether patient had changed room during ICU stay; *p* values for the difference between intervention and control rooms are based on ordinal probit models with factors in SMB as dependent variable, controlled for the same variables as the weighted mean values.

## Results

A total of 99 observations were included in this study, of which 69 were from one of the two control rooms and 30 were from the intervention room (Table 4). There were no significant differences between the characteristics of the visitors in the control rooms and the intervention room regarding sex, age, number of visits, and relationship to the patient; likewise, there was no difference in whether the patient had changed patient room during the stay at the ICU (Table 4).

### 

#### The PCQ-F

Ordinal probit models were used to estimate the difference between the control and intervention rooms in the variables concerning the ward climate. Regression was executed in three different models (see Table 1).

The visitors who visited critically ill patients in the intervention room had a significantly more positive scoring in their perceptions of the psychosocial ward climate than those visiting in the control rooms did.

***The visitors who visited critically ill patients in the intervention room had a significantly more positive scoring in their perceptions of the psychosocial ward climate than those visiting in the control rooms did.***

The visitors who visited critically ill patients in the intervention room had a significantly more positive scoring in their perceptions of the psychosocial ward climate than those visiting in the control rooms did (Table 1). Nevertheless, when assessing the ward climate concerning the staff, there were no significant differences between the control rooms and the intervention room (Table 1).

#### The SMB

Linear regressions were used to obtain adjusted mean values for the different dimensions for the intervention and control rooms, respectively (Please see figure 4). Ordinal probit models were then used to study whether there were significant differences between the intervention and control rooms (see Figure 1).

The results for the SMB showed significant differences favoring the intervention room on the factors of complexity and pleasantness with borderline significance on the modern factor (see Table 3).

## Discussion

This study examined different features of the healthcare environment. Both PCQ-F and SMB were used for the first time in an ICU context. Using the PCQ-F, the results showed the intervention room was significantly perceived as having both a better ward climate in general and greater everydayness than the control rooms did. This indicates that the visits in the refurbished environment in this high-tech context represented a more positive experience. For the families and friends visiting the intervention room, this meant that the staff were perceived as accessible, amenable, competent, and comprehensible. It also meant that the room was viewed as more familiar, offering peacefulness and a positive distraction from illness by having something beautiful to look at during the visits (J. Lindahl et al., 2015). Since the staff in this study were not allocated to only one of the patient rooms at the ICU, but instead worked in all the patient rooms, the result concerning the staff was not surprisingly different between the differently designed patient rooms. The SMB results showed significant perceived differences benefiting the intervention room on complexity and pleasantness with borderline significance on modern by the visitors. Previous studies have found that when pleasantness was perceived as high, the environment was also perceived as safe, secure, and stimulating (Bengtsson & Carlsson, 2006; Shih & Ramilo, 2014).

Previous research has reported that families of critically ill patients cared for in ICUs experience serious types of ill-being such as depression, anxiety, and fatigue (Apple, 2014; Celik et al., 2016; Day et al., 2013); sometimes, they even develop post-traumatic stress disorder (Petrinec & Daly, 2016; Stayt & Venes, 2019; Wintermann et al., 2016). These findings from the refurbished intervention room can be a way of reducing some elements of ill-being. EBD aims at implementing various research disciplines into healing environments (Hamilton & Watkins, 2009), and this study strengthens and contributes to that theory/idea; that is, the study shows that it is possible to design and build for better health and well-being.

The instrument of PCQ-F is rooted in person-centered care. PCC aims to see the person behind the patient, so does caring science. The focus on the patient also includes the recognition of the whole family since PCC and caring science aim to provide healthcare that is humble and responsive to individual families’ needs and beliefs (Davidson et al., 2017; Gerritsen et al., 2017). The results of this study showed that the visiting family members scored the intervention room as being more pleasant, less complex, and more familiar (everydayness). This indicates that when visiting an enriched healthcare environment at an ICU and experiencing a less alien environment that could reduce the amount of stress usually experienced by family members of critically ill patients. The findings of Ulrich et al. (2019) support this statement as they found that family members who had a natural scenery had less amount of stress than those who did not have access to nature or other positive distractions.

By designing and constructing enriched healthcare facilities, especially in the intensive care context, where there is an extra dimension of saving lives, this study facilitates increased health and wellbeing of the patients’ visitors.

***By designing and constructing enriched healthcare facilities, especially in the intensive care context, where there is an extra dimension of saving lives, this study facilitates increased health and wellbeing of the patients’ visitors.***

By designing and constructing enriched healthcare facilities, especially in the intensive care context, where there is an extra dimension of saving lives, this study facilitates increased health and well-being of the patients’ visitors. Previous research has shown that this also improves the well-being of the staff in intensive care settings (Sundberg et al., 2017). The whole human being is far more complex than its parts are. The same is true of the healthcare environment, where the wholeness can be termed *atmosphere*, defined as “a surrounding influence or environment” (Merriam-Webster, 2020). Atmosphere is a synonym of climate, which was used in this study via the PCQ-F’s term, *ward climate*. The design of healthcare facilities plays a crucial part in not only the built environment but also the lived environment, the atmosphere. Today, many of these healthcare facilities are constructed to enhance clinical efficiency. This may cause great risks for depersonalization, but the trend has changed toward designing more person-centered facilities today, and this often increases stakeholders’ well-being (McCormack et al., 2011). An aspect of comfort is linked to the surrounding environment (Olausson et al., 2019): It is even possible to experience at-homeness in such high-technology settings as ICUs when the design matches the needs of the patients, their family, and the staff (Andersson et al., 2019).

It is even possible to experience at-homeness in such high-technology settings as ICUs when the design matches the needs of the patients, their family, and the staff.

***It is even possible to experience at-homeness in such high-technology settings as ICUs when the design matches the needs of the patients, their family, and the staff.***

EBD aims to create healing environments, as does caring science. Therefore, a match between these research fields is of great interest, and more successful collaborations are needed in the future as these disciplines have the same goal—to ensure persons in healthcare facilities have the highest possible well-being.

## Limitations

Collecting data is not always in the control of the researchers as in this study where nursing staff helped to distribute the questionnaires. There was a potential for bias in who was chosen to participate in this survey. However, this study would not have been possible if the researchers had handled the questionnaire distribution since none were employed at this ICU, and the participants needed to answer the questionnaires while being in the specific patient rooms. Thus, the data collection, abiding also by ethical constraints, was allocated to the nurses working in that ICU. Another limitation of the study was the small sample size. Despite the long period of data collection, only 99 questionnaires were included in this study. This is likely connected to the fact that the researchers did not have control over the data collection process as well as the vulnerability of the potential participants who were focused on loved ones in a critical situation rather than participating in research.

A critique of the SMB questionnaire is that it may be obsolete because it was developed in the 1960s and 1970s. This may relate to the outcome in this study. Semantics encompasses the meaning of language and significations of words (Merriam-Webster, 2020); since language develops at the same pace as society, this questionnaire, the words it uses, and even their meanings now may seem outdated. Therefore, an updated version may have been in place. However, there are few questionnaires concerning the semantics of the built environment.

## Implications for Practice

- The healthcare environment in ICUs may be perceived as overwhelming and increase visitors’ stress levels. Architects and designers should consider arranging indoor and outdoor settings so that they will be perceived as safer and having everydayness, encompassing stress-reduced positive distractions. The findings suggest that an enriched healthcare environment in critical care can be an effective intervention to create safety, a less hospital-like setting, and greater homeliness in the atmosphere.
- The study implies that, despite living through a time in which a close relative or friend is experiencing a life-threatening condition, visitors are aware of the surrounding environment. Therefore, the importance of the built environment for health and well-being should be on every stakeholder’s agenda.
- Multidisciplinary teams need to collaborate when planning for new construction or refurbishment of intensive-care settings. By incorporating the competences of architects, designers, nurses, former patients, and patients’ family members, those with expertise can work together, and every aspect can be considered to provide the best possible outcome for every stakeholder included.
